# Radiotherapeutic Modalities and Advancements in the Treatment of Cutaneous Malignancies

**DOI:** 10.3390/jcm14186547

**Published:** 2025-09-17

**Authors:** Noor Malik, Irini Yacoub, Kristin Hsieh, J. Isabelle Choi, Arpit Chhabra, Charles B. Simone

**Affiliations:** 1 School of Medicine and Dentistry, University of Lancashire, Preston PR1 2HE, UK; 2 New York Proton Center, New York, NY 10035, USA; 3 Department of Radiation Oncology, Memorial Sloan Kettering Cancer Center, New York, NY 10065, USA; 4 Department of Radiation Oncology, Mount Sinai Medical Center, New York, NY 10029, USA

**Keywords:** cutaneous malignancies, skin cancer, non-melanoma skin cancer, malignant melanoma, radiotherapy, brachytherapy, electron beam therapy, proton therapy, immunotherapy

## Abstract

Cutaneous malignancies represent the most common cancers worldwide and pose a growing public health burden. While surgical excision remains the primary curative modality, radiotherapy offers an effective adjuvant therapy for high-risk histopathologic features and an established, organ-preserving alternative for patients with inoperable disease or lesions in cosmetically or functionally sensitive sites. Advances in radiotherapeutic techniques, including brachytherapy and proton therapy, have expanded the therapeutic armamentarium, allowing tailored treatment based on tumor depth, extent, and anatomical location. Contemporary evidence highlights favorable local control and toxicity outcomes with modern radiation therapy approaches, yet data remain fragmented, with most studies limited by small cohorts, heterogeneous methodologies, and limited follow-up durations. Furthermore, the role of radiotherapy in complex scenarios, such as perineural invasion, recurrent disease, and previously irradiated fields, continues to evolve. This review synthesizes the current literature on radiotherapeutic management of skin cancer, critically evaluates dosimetric and clinical outcomes across modalities, and identifies key gaps in evidence. Emphasis is placed on the need for prospective, multicenter investigations to better define comparative effectiveness, optimize dose-fractionation regimens, and integrate emerging technologies into clinical practice. Radiotherapy remains an indispensable modality in dermatological oncology, offering curative potential with preservation of cosmesis and function, yet its optimal utilization demands further high-quality research to refine patient selection and therapeutic strategies.

## 1. Introduction

Cutaneous malignancies constitute the most prevalent cancer type globally [1,2]. Epidemiologic data reveal that over 5.4 million new cases of non-melanoma skin cancers (NMSCs) are diagnosed annually in the United States alone [3], with the incidence rising at an estimated rate of 2–3% per year [4]. The pathogenesis of skin cancers is primarily driven by the cumulative exposure to ultraviolet (UV) radiation, which induces unrepaired DNA damage in epidermal cells, thereby facilitating oncogenic mutations and uncontrolled cellular proliferation [5]. The skin is structurally composed of two principal layers: the superficial epidermis, consisting predominantly of keratinocytes and melanocytes, and the underlying dermis, which comprises vascularized connective tissue containing adnexal structures such as sweat glands and hair follicles [6]. Malignant transformation of melanocytes gives rise to malignant melanoma (MM), a relatively rare but highly aggressive neoplasm characterized by a predilection for metastatic spread and a high risk of recurrence, particularly when arising from dysplastic or pre-existing nevi [7,8]. Despite accounting for a smaller proportion of total cutaneous malignancies, MM is responsible for most skin cancer-related deaths, with an estimated 8000 to 9000 deaths annually [9]. In contrast, the vast majority of skin cancers originate from the keratinocyte lineage and present as cutaneous basal cell carcinoma (cBCC) or cutaneous squamous cell carcinoma (cSCC). These non-melanoma subtypes typically exhibit more indolent behavior with favorable survival outcomes when detected early [10,11,12]. However, delays in diagnosis or high-risk histological features may result in local tissue invasion and, in a minority of cases, regional or distant metastasis. In addition to these common malignancies, rare skin cancers—including Merkel cell carcinoma (MCC), cutaneous adnexal tumors, and cutaneous lymphomas—pose unique diagnostic and therapeutic challenges. Although uncommon, these subtypes can exhibit more aggressive behavior [13,14] and, thus, require careful clinical evaluation.

Surgical excision remains the first-line intervention for most localized cutaneous tumors, offering high curative potential with complete resection [15]. However, incomplete resection due to subclinical tumor extension can increase the risk of local recurrence [16], particularly in tumors exhibiting high-risk histopathological features, such as perineural invasion (PNI), lymphovascular spread, or regional nodal involvement [17,18,19]. Additionally, a surgical approach may be suboptimal in patients with significant comorbidities or disease located in anatomically sensitive regions, where extensive excision may result in functional compromise or disfigurement [20]. In 2021, the European Association of Dermato-Oncology (EADO) introduced a novel operational staging system for cBCC [21], followed by updated European consensus guidelines for cSCC [22], published in 2023, covering diagnostics, prevention, and treatment. These frameworks refine risk stratification and therapeutic pathways and emphasize the expanding role of radiotherapy (RT), particularly in cases where cosmetic and functional preservation are important. Additionally, many patients with skin cancer are older adults and may have multiple comorbidities [23]. Factors such as vascular insufficiency, fragile or atrophic skin, and impaired wound healing may limit surgical options [23], making non-invasive approaches especially appealing. RT is also particularly beneficial for immunocompromised patients [24], as it provides effective local tumor control without the increased infection risk or systemic immunosuppressive effects associated with surgery and systemic therapies. In this context, RT emerges as a key component of the multidisciplinary treatment paradigm, serving both as a definitive modality for patients unsuitable for surgery and as an adjuvant strategy in surgical patients to improve locoregional control. Beyond its curative intent, RT also plays a vital role in palliation by offering durable local symptom relief, pain control, and improved quality of life in advanced or unresectable cases. This review delineates the evolving role of RT in the management of cutaneous malignancies, emphasizing conventional photon-based techniques, electron beam therapy, brachytherapy (interventional RT) (BT), and proton beam therapy (PBT). Importantly, several key insights in this review update and expand upon existing literature. First, it incorporates the most recent advances up to 2025, ensuring up-to-date evidence for clinical decision-making. Second, it presents updated clinical results with eBT and PBT, including locoregional control and cosmetic outcomes. And finally, it discusses emerging modalities that have influenced skin cancer management recent decades, such as neutron therapy, and the selective integration of immunotherapy in select cases, highlighting evolving multimodal management strategies for skin cancer. Due to the limited availability of higher-level evidence, the data presented in this review are primarily based on Level IV evidence from retrospective cohort analyses.

## 2. Methodology

A literature search was conducted using PubMed and Google Scholar databases. The following keywords and combinations were used: cutaneous malignancies, skin cancer, non-melanoma skin cancer, malignant melanoma, Merkel cell carcinoma, radiotherapy, superficial X-ray therapy, orthovoltage X-ray therapy, three-dimensional conformal radiotherapy, intensity-modulated radiotherapy, volumetric modulated arc therapy, brachytherapy, electron beam therapy, proton therapy, immunotherapy. Articles published in English were considered, with preference given to studies focusing on clinical outcomes, dosimetric comparisons, toxicity profiles, and cosmetic results. Additional references were identified from the bibliographies of relevant articles.

## 3. Consensus Recommendations and Evidence for Radiotherapy in Cutaneous Malignancies

Building on the previously described role of surgery and the general principles of RT in cutaneous malignancies, the following section provides a detailed, evidence-based synthesis of RT applications across tumor types, treatment intents, and modalities [17,18,19,25,26,27,28]. This includes definitive primary therapy for unresectable or functionally sensitive lesions, adjuvant therapy for high-risk or margin-positive tumors, nodal irradiation for selected high-risk cases, and palliative treatment for symptom control in advanced disease. The discussion and accompanying table highlight guideline-supported modalities—such as superficial/orthovoltage X-rays, electron beams, high-dose-rate BT (HDR-BT), eBT, and advanced photon techniques (three-dimensional conformal RT (3D-CRT), intensity modulated RT (IMRT), volumetric modulated arc therapy (VMAT))—as well as PBT in anatomically challenging sites. Emerging approaches, including boron neutron capture therapy (BNCT) and carbon-ion therapy, are currently investigational but highlight ongoing innovation in the field. Where available, toxicity outcomes are reported using standardized grading systems such as the Radiation Therapy Oncology Group (RTOG) acute and late toxicity scales [29], as well as the Common Terminology Criteria for Adverse Events (CTCAE) [30], to ensure consistency in reporting and facilitate cross-study comparisons. By organizing the data according to treatment intent and technical approach, the following table (Table 1) aims to provide a practical, clinically relevant framework for optimizing tumor control while minimizing toxicity. 

## 4. Kilovoltage X-Rays

### 4.1. Superficial X-Ray Therapy

Superficial X-ray therapy (SXRT) stands amongst the earliest treatment modalities for skin cancer and has long been integral to dermatological cancer care [31]. Despite a decline in use over recent decades, largely due to the rise in Mohs micrographic surgery and the limited availability of modern SXRT units [32], SXRT continues to be employed in select clinical settings where it provides specific advantages, including in regions with limited access to advanced RT options. SXRT delivers low-energy kilovoltage photons, typically 50–100 kVp, to lesions with tissue depths up to 2 mm. Surface applicators or contact cones are placed directly over the lesion. The dose is highest at the skin surface and decreases exponentially with depth [33], allowing effective targeting of superficial tumors. Coupled with a minimal lateral penumbra of around 1 mm [34], these characteristics enable precise treatment of well-defined skin lesions in cosmetically and functionally sensitive areas, although its use is limited on highly irregular surfaces. In clinical practice, SXRT is commonly used for lower-extremity lesions, including below-the-knee sites, where surgical wound healing is often delayed [34]. In this anatomically challenging context, it may better prevent postoperative complications such as cellulitis compared to surgery, making it particularly valuable for elderly or medically frail patients with impaired healing capacity [34].

Treatment regimens are tailored according to tumor size, histology, and patient comorbidities [35]. More moderately hypofractionated schedules are especially advantageous for frail or elderly patients, reducing the overall treatment burden without compromising efficacy. The technical setup for SXRT is relatively straightforward, employing a short source-to-surface distance (SSD) and fixed applicators. Compared to megavoltage RT, the shielding requirements are simpler and more localized, as lower-energy photons do not necessitate large-scale room or gantry shielding [34]. Nevertheless, custom-fabricated lead shielding remains essential for all patients to ensure precise beam collimation and prevent inadvertent contact between the radiation cone and surrounding tissue, which may result in undertreatment [36].

Clinical outcomes for NMSC with SXRT are excellent. Five-year LC rates frequently exceed 90% [36]. For instance, in a large retrospective study by Cognetta et al. [37], 1715 NMSCs treated with 5 fractions of 7 Gy (total 35 Gy) or with 7 fractions of 5 Gy (total 35 Gy) for lip lesions, reported cumulative LC rates of 98.1% at 2 years and 95% at 5 years. Schulte et al. [38] published an earlier retrospective study of 1267 NMSCs, determining a 5-year cure rate of 93.8%. The average total radiation dose was 61 Gy for cBCCs and 63.6 Gy for cSCCs. Tumors treated two or three times per week received an average dose of 61.5 Gy, while those irradiated six times per week received 61.4 Gy. Modern devices have reinvigorated the interest in SXRT by offering compact, user-friendly platforms with real-time imaging integration and dosimetric flexibility [39]. A multi-institutional analysis of 3050 NMSC lesions treated with image-guided SXRT (IGSXRT) achieved an absolute LC rate of 99.2% in 3027 lesions at a median follow-up of 25.1 months, with no disease-specific mortality and consistently excellent cosmetic outcomes [40]. The incorporation of dermal ultrasound in IGSXRT systems enables more accurate depth targeting and margin definition, thereby minimizing RT exposure to healthy tissue [41]. Nevertheless, limitations remain, particularly in cases of subclinical extension or thicker lesions, where accurate assessment of depth may be difficult without imaging or surgery. SXRT has also shown high efficacy for minimal stage IA Mycosis Fungoides [42]. A series of 21 patients with 32 lesions, treated with either low-energy X-rays or 4–12 MeV electrons, demonstrated a complete response rate of 97%, with all patients alive at last follow-up (median 36 months, range 13–246 months), and treatment was generally well-tolerated with only mild skin reactions reported. In MCC, superficial and orthovoltage X-ray therapy has similarly demonstrated substantial efficacy [43]. In 67 patients, 62 stage I-III cases treated with radical intent were analyzed (median age 74 years). Among 42 stage I-II patients, those receiving RT to the primary site achieved a 2-year local recurrence-free survival of 89% versus 36% for patients not receiving RT (*p* < 0.001). Cumulative 2-year regional recurrence-free survival for patients receiving adjuvant regional RT was 84% versus 43% for those who did not (*p* < 0.001). Immune status at initial surgery was a significant predictor of overall survival, independent of tumor size. More recent evidence on Mycosis Fungoides and MCC is largely limited to small case reports, highlighting that while SXRT remains a potential option, contemporary large-scale studies are lacking. 

Overall, while not currently emphasized in all major guidelines, SXRT continues to be utilized in selected centers and medical offices worldwide, and offers a compelling blend of efficacy, safety, and patient convenience.

### 4.2. Orthovoltage X-Ray Therapy

Orthovoltage X-ray therapy (100–300 kVp) extends the treatment capabilities of SXRT within the kilovoltage external beam spectrum. While SXRT is generally used for lesions confined to the epidermis and superficial dermis, orthovoltage beams allow effective treatment of thicker tumors. A 100 kVp energy beam with a half-value layer (HVL) of 7 mm aluminum deliver nearly full dose at the surface, approximately 85% at 5 mm depth, and around 70% at 10 mm, making them suitable for lesions up to 5 mm deep [44]. Higher-energy beams (200–300 kVp), filtered with 2–4 mm copper, maintain roughly 95% of the surface dose at 5 mm depth and ~90% at 1 cm, enabling coverage of lesions approaching 1 cm in thickness [44]. These depth-dose characteristics are particularly useful for tumors with microscopic extension beyond the superficial dermis.

Compared to electrons, orthovoltage fields demonstrate broader lateral dose coverage at depth; for instance, the 95% isodose width for a 3 cm circular field is approximately 32% greater than that of 6 MeV electrons, thereby improving coverage of perilesional microscopic spread without the need for extensive field expansion [45]. However, orthovoltage X-rays are less suitable for highly irregular surfaces, and photon absorption in bone is more pronounced due to the increased photoelectric effect at lower photon energies [46]. This may lead to localized hotspots over convex bony structures such as the forehead or scalp [46], necessitating careful planning.

The technical setup is like that of SXRT, using fixed applicators, short source-to-surface distances (SSD), and minimal shielding requirements, which allows for efficient outpatient workflows. However, beam collimation and shielding are often less conformal compared to modern modulated techniques [24]. Nonetheless, in selected cases, particularly in elderly or frail patients, orthovoltage X-rays offer excellent LC rates (>90%) [47], low toxicity, and favorable cosmetic outcomes, especially when delivered with short-course, hypofractionated regimens. This is further supported by the large single-institutional series by Marconi et al. [48], who retrospectively analyzed 1021 head and neck NMSC lesions treated with orthovoltage X-ray therapy. They reported 5- and 10-year LC rates of 96% and 94% for cBCC and 92% and 87% for cSCC, respectively, demonstrating superior outcomes for cBCC compared with cSCC. Importantly, they found that fractionation schemes delivering >2.0 Gy per fraction were associated with improved LC on multivariate analysis, without increased toxicity, supporting the rationale for hypofractionated regimens. Similar approaches have been explored in superficial melanomas, particularly lentigo maligna melanoma (LMM), which is predominantly superficial in nature. A review of RT techniques for lentigo maligna (LM) and LMM found that 36% of cases were treated with superficial or orthovoltage X-rays, demonstrating their role in select patient populations [49]. In a series of 64 patients with LM and LMM that were treated with 100 Gy of orthovoltage RT in 10 fractions, only two local recurrences occurred at 13 and 44 months, both successfully salvaged surgically, highlighting excellent LC with low toxicity [50]. Beyond the clinical efficacy, patient-reported outcomes consistently favor orthovoltage X-ray therapy. Kharofa et al. [51] reported that 94% of patients (*n* = 42) treated with orthovoltage X-rays for facial NMSCs were “satisfied” or “very satisfied” with their cosmetic outcomes, with Skin Cancer Index quality-of-life (QoL) scores comparable to those observed following surgery.

Overall, orthovoltage X-ray therapy complements SXRT by extending the treatable depth range while maintaining high LC rates, low toxicity, and logistical simplicity, making it a valuable option for appropriately selected cases. 

An overview of key studies evaluating kilovoltage X-ray therapy outcomes is presented in Table 2.

## 5. Three-Dimensional Conformal Radiotherapy

3D-CRT utilizes CT-based planning to shape radiation dose distributions that conform to the geometry of the target volume [57]. Compared to the older 2D approaches, 3D-CRT allows for more precise tumor targeting through beam’s-eye-view planning, multiple fixed-angle beam arrangements, and anatomical contouring of both target and normal structures [58]. This improves dose homogeneity within the clinical target volume (CTV) and minimizes radiation exposure to surrounding normal tissues, particularly critical organs [57]. A dosimetric study [59] comparing 2D-CRT, 3D-CRT, intensity-modulated RT (IMRT), and VMAT for regional nodal irradiation in patients with locally advanced or high-risk skin cancers demonstrated that 3D-CRT provided substantial improvements over 2D-RT in terms of dose homogeneity and sparing of several organs at risk (OARs), especially in the groin and axilla. Clinically, the Australia and New Zealand Melanoma Trials Group (ANZMTG) 01.02/Trans-Tasman Radiation Oncology Group (TROG) 02.01 phase III trial showed that adjuvant 3D-CRT significantly decreased in-field recurrence by 50% in patients with macroscopic regional nodal melanoma, delivering 48 Gy in 20 fractions, highlighting its effectiveness in high-risk nodal disease [60]. While IMRT and VMAT can further reduce doses to surrounding healthy structures such as the humerus, brachial plexus, bladder, and femur [59], 3D-CRT remains a practical and effective option in resource-limited settings or when advanced techniques are not required due to lesion size, depth, or anatomical considerations. Because it lacks the intensity modulation capabilities of IMRT and VMAT and may involve increased integral dose from larger treatment fields [59], 3D-CRT has largely been replaced in modern practice for anatomically complex or deeply located lesions.

## 6. Intensity-Modulated Radiation Therapy

IMRT enables highly conformal dose distribution by modulating of the intensity of individual beamlets across multiple radiation fields [61]. Its ability to modulate beam intensity from multiple angles allows precise coverage of tumors with complex geometry, while its deep penetration enables treatment of lesions extending beyond superficial tissues [61]. Using inverse treatment planning algorithms and CT-based volumetric imaging, IMRT achieves superior sparing of adjacent normal tissues compared to conventional 2D or 3D-CRT approaches [61], which is beneficial particularly for tumors situated near critical structures. This makes IMRT especially suitable for the anatomically complex situation and in the adjuvant or recurrent setting [62,63,64].

Evidence supporting the clinical application of IMRT in advanced NMSC and MM is gradually emerging. A retrospective study of 21 patients with stage T4 NMSCs treated with various RT modalities between 2004 and 2010 included a subset managed with IMRT, yielded promising results [65]. Specifically, 60% of patients treated with IMRT achieved LC following definitive RT alone, and 80% remained disease-free after subsequent surgical salvage treatment. Outcomes were superior in patients receiving IMRT as primary or adjuvant therapy compared to those treated for recurrent disease, and they were further superior in individuals with cBCC histology, and without bone or nodal involvement. Similarly, a retrospective review of 46 patients with head and neck MM and regional lymph node metastases treated with lymphadenectomy and adjuvant IMRT demonstrated favorable locoregional control and acceptable toxicity [66]. Adjuvant IMRT was delivered twice weekly over 2.5 weeks to a total dose of 30 Gy in 5 fractions. Most patients experienced only grade 1-2 acute dermatitis and mucositis, with no grade ≥3 late adverse events. Three-year planning target volume (PTV) and total locoregional control rates were 85% and 76%, respectively, with overall survival and disease-free survival of 63% and 25%. Notably, 23 patients in this cohort were treated with either IMRT or 3D-CRT, illustrating the practical applicability of conformal RT in high-risk or recurrent settings. Collectively, these studies highlight the utility of IMRT in achieving meaningful locoregional control while emphasizing careful patient selection, particularly in cases with adverse risk features such as nodal metastases or prior recurrence. Nevertheless, the clinical application of IMRT presents several limitations. While its deep penetration allows treatment of thick or infiltrative lesions, superficial dosing may be insufficient without the use of a tissue-equivalent bolus [67], complicating setup and reproducibility. Additionally, IMRT involves greater resource usage, prolonged treatment planning, and longer delivery times, all of which may be less feasible in elderly, frail patients or less practical when treating small, indolent lesions [58]. Moreover, the broader low-dose bath (uniform, low-dose irradiation of a large tissue or skin area) inherent to IMRT raises concerns about increased integral dose [68], although the clinical significance of this remains limited in the older NMSC demographic. While the theoretical advantages of IMRT are notable, current clinical evidence remains largely retrospective and institution-specific. Thus, further prospective studies are warranted to better define its role, particularly in the context of high-risk and anatomically complex cutaneous malignancies.

## 7. Volumetric Modulated Arc Therapy

VMAT is an advanced form of IMRT that delivers highly conformal radiation by modulating beam shape, dose rate, and gantry speed during continuous RT delivery [69]. This allows for efficient, precise dose delivery with reduced treatment times compared to conventional IMRT [70]. Its high degree of conformality and ability to create sharp dose gradients make it especially advantageous for treating large, irregular, or anatomically complex cutaneous malignancies, particularly in the head and neck region, where critical OARs lie in close proximity, allowing optimal tumor coverage while sparing OARs [71]. VMAT is increasingly favored for re-irradiation and is commonly used for tumors that are deeply infiltrative, advanced-stage, or have perineural tumor spread, as well as postoperative cases with close or positive surgical margins. Some commonly reported side-effects include alopecia, telangiectasia, dryness, erythema, and ulceration, especially when treating scalp or facial lesions [72].

Clinical data, while still limited, are emerging. Existing studies have shown VMAT to be effective in delivering complex plans with favorable LC and cosmesis. A retrospective cohort study [73] of patients with unresectable head and neck cSCC treated with upfront radiation therapy in Sydney demonstrated an objective response rate of 97%, with higher biologically equivalent doses (≥60 Gy) associated with improved infield progression-free survival (78% at 6 months) and overall survival (65% at 24 months), highlighting the importance of dose escalation in achieving durable control in frail patients. Similarly, an institutional series [74] of definitive RT for patients with advanced inoperable cSCC, in which IMRT/VMAT was used in 8 (28%) patients, reported a median overall survival of 21 months and a 5-year cumulative incidence of progression of approximately 46%. Nearly three-quarters of tumors responding to RT remained progression-free at 5 years, even among patients with in-transit or nodal metastasis. A case series [75] evaluating VMAT for extensive skin field cancerization involving 32 patients demonstrated >90% clinical clearance in 87% of cases, with no grade ≥3 toxicities reported at 12-month follow-up. This highlights the potential role of VMAT in diffuse disease. The application of jaw tracking during VMAT planning has also been shown to significantly reduce the volume of low-dose radiation delivered to surrounding tissues without compromising target coverage. In a study [76] of 50 patients with facial NMSC, jaw tracking reduced the volume of low-dose volumes, defined as the volumes receiving <50% of the prescribed dose in this study (V10-50%). This may potentially lower radiation exposure to healthy tissue and improve cosmetic outcomes, although more research is needed to confirm its clinical benefits.

The main concern with VMAT, as with IMRT, is the potential for skin underdosing due to the skin-sparing nature of megavoltage photons [77]. Careful application of bolus material is essential to ensure adequate surface dosing, especially for shallow lesions. There is also the potential for low-dose radiation “bath” to a larger volume of normal tissue compared to static field techniques [78], although its clinical significance in skin cancer remains under investigation. Additionally, VMAT requires advanced treatment planning systems, rigorous quality assurance, and sophisticated delivery technology, which may not be readily available in some clinical settings [79]. The planning process involves contouring the target and OARs on CT images, defining the prescription dose, selecting rotational arcs, and optimizing beam fluence and gantry speed using inverse planning. Key parameters, including collimator angle, dose rate, and modulation, are adjusted to ensure optimal target coverage while sparing OARs, and tools such as jaw tracking and avoidance sectors are employed to minimize low-dose exposure. Plan quality is confirmed through DVH (dose-volume histogram) analysis and pre-treatment phantom verification. Overall, whilst not first-line for small or purely superficial lesions, VMAT represents an important option for complex or high-risk cutaneous malignancies, particularly when optimal conformality is required. Emerging evidence supports its efficacy as a definitive modality in unresectable or advanced disease.

## 8. Electron Beam Therapy

Electron beam therapy remains a clinically relevant and widely used modality in the management of cutaneous malignancies. Electron beams offer high surface dose distribution to superficial tissues with a sharp distal fall-off [80], making them suitable for lesions confined to the skin and superficial subcutaneous tissue, while still penetrating deeper than kilovoltage X-rays (up to ~5 cm depending on beam energy) and sparing underlying structures [81]. Treatment energies typically range from 6 to 20 MeV, with the therapeutic depth corresponding to the 90% isodose line [82]. Electrons penetrate superficially and resulting in less skin-sparing than megavoltage photons [83]. To ensure adequate surface dosing, treatment planning involves selecting appropriate beam energy, field size, and bolus thickness, often guided by CT-based simulation or 3D planning for irregularly shaped lesions. Customized cutouts or electron-specific collimation devices are frequently used to improve dose conformity. A bolus (e.g., paraffin wax) is often applied to shift the dose distribution closer to the skin surface, which thickness adjusted based on beam energy and lesion depth.

Several limitations remain with electron beam therapy. Due to the scattering characteristics of electrons, the beam profile can constrict at both the surface and depth, leading to underdosage in peripheral target regions, particularly in small or irregularly shaped fields. To compensate, field expansion by approximately 1–1.5 cm is often required [82], or the treatment planning is adjusted accordingly. Amdur et al. [45] demonstrated that, in a 3 cm field, the 95% isodose width was approximately 32% greater with orthovoltage X-rays compared to 6 MeV electrons, thus emphasizing the need for generous margins in electron planning. The use of customized cutouts or skin-contact collimation devices can improve conformity but adds to planning and delivery complexity. Electron dosimetry is highly sensitive to block geometry and output factors, requiring rigorous quality assurance, including depth-dose measurements, output calibration, and verification of field uniformity [84]. Additionally, lateral scatter and reduced side-scatter equilibrium contribute to outward bowing of the low-dose isodose lines, which broadens the penumbra and may increase dose to surrounding normal tissues [85]. Collectively, these factors complicate treatment planning, prolong setup time, and may reduce patient comfort.

Despite these technical considerations, electron beam therapy remains an effective option for appropriately selected lesions. Literature reports for patients with localized skin cancers treated with electron beams indicate LC rates around 88%, with overall control reaching 93% when recurrences were salvaged with surgery or additional RT. Cosmesis is generally excellent or good in over 90% of patients, and side effects are typically mild and self-limiting [86]. In more diffuse cutaneous malignancies, such as cutaneous T-cell lymphoma, total skin electron beam therapy (TSEBT) demonstrates high overall response rates with both low dose (12 Gy) and standard dose (35 Gy) regimens, along with favorable short-term safety profiles. One study [87] retrospectively analyzed 51 patients treated with TSEBT (31 received 35 Gy, 20 received 12 Gy) with dose selection based on the extent of skin involvement. Results showed a median time to meaningful progression of 5.1 months and overall survival of 27.4 months, with both measures significantly better in T2 versus T3 stage. The overall response rate was 80.4%, symptom improvement occurred in both dose groups, and treatment was generally well tolerated. If whole-body electron therapy is unavailable, whole-body photon techniques such as VMAT or helical tomotherapy may be considered as alternatives. These findings highlight the versatility and clinical value of electron-based therapies, from localized skin cancers to diffuse cutaneous lymphomas, emphasizing their efficacy, safety, and cosmetic advantages.

## 9. Brachytherapy

BT offers a highly conformal treatment option for cutaneous malignancies, particularly in elderly or surgically ineligible patients, and is especially useful for lesions on anatomically irregular, curved, or complex surfaces [88], where conventional techniques may struggle to achieve uniform dose coverage. The development of HDR-BT afterloading techniques and electronic brachytherapy (eBT) has renewed interest in BT, especially for definitive treatment. Treatment is typically delivered via interstitial catheters or customized surface molds, though other modalities such as low-dose-rate BT (LDR-BT) and pulse-dose-rate BT (PDR-BT) may also be employed, each with distinct physical and clinical properties.

Overall, BT achieves excellent LC and favorable cosmetic outcomes for well-defined lesions, although limitations include technical complexity, resource requirements, and the need for meticulous planning and imaging to ensure accurate dose delivery.

### 9.1. High-Dose-Rate Brachytherapy

HDR-BT is typically administered using a remote afterloading system with an Iridium-192 (Ir-192) source and encompasses both superficial and interstitial techniques. Superficial BT is generally used for lesions up to 5 mm in depth; however, recent evidence supports treating thicker lesions using multilayer catheter arrangements. Image guidance is essential to accurately determine lesion depth and define the CTV, ensuring adequate coverage while minimizing unnecessary toxicity [89]. For lesions exceeding 5 mm, interstitial or multilayer catheter approaches enable dynamic, intensity-modulated dose delivery, which optimizes CTV coverage and helps expand the therapeutic window—the distance between the prescribed dose (100% isoline) and the maximum tolerable dose to normal tissues (often up to 150% isoline in skin BT) [89]. By adjusting catheter-to-skin distances and layering catheters, the therapeutic window can be maximized, ensuring high tumor dose while sparing surrounding skin [89]. HDR-BT utilizes standardized applicators such as Leipzig and Valencia devices for flat or shallow lesions, as well as flexible catheter arrays, such as Freiburg flaps, which contour slightly curved surfaces. Custom-made thermoplastic molds, often generated using 3D printing from materials like resin, wax or alginate, allow for individualized dose delivery in anatomically complex regions such as the nose or ear [90]. It typically requires local or regional anesthesia, and thin-slice CT imaging (≤2 mm) is used for catheter and target delineation.

Dosimetrically, HDR-BT achieves a steep dose gradient, with approximately a 50% dose reduction within 1 cm, permitting tight treatment margins (~0.5–1 cm) and quantitative sparing of adjacent normal tissues [91,92]. Compared with conventional EBRT, HDR-BT and eBT enable precise dose escalation within the target volume, rapid dose fall-off at the tumor periphery, optimal protection of adjacent sensitive structures, shorter treatment times, and compatibility with hypofractionated schedules. Using inverse planning, source dwell times and positions are optimized to sculpt the dose precisely, with typical regimens delivering 40–50 Gy over 8 to 10 fractions, depending on tumor size and histology. While HDR-BT achieves excellent conformity, its lack of continuous low-dose exposure (unlike LDR-BT or PDR-BT) may reduce the radiobiologic benefit of normal tissue repair between fractions [90].

Clinical studies report excellent LC rates ranging from about 83% to nearly 100%, with HDR-BT being well tolerated across various applicator types and schedules. One of the earliest large-scale data came from Köhler-Brock et al. (1999) [93], reporting a 91% LC rate in 520 lesions treated with the Leipzig applicator. Delishaj et al. [94] later reported 96.25% complete response using the Valencia applicator on 45 lesions, with no grade 3 or higher acute or late toxicities after 47 months of follow-up. Findings from the SCRiBe meta-analysis [95] further support HDR-BT’s effectiveness. Among 553 patients treated with BT versus 9965 patients treated with EBRT for stage T1-2N0 NMSCs, both modalities showed <7% local recurrence at 1 year (median dose of 45 Gy in 10 fractions). However, BT yielded significantly better cosmetic outcomes, demonstrating 95% “good” cosmesis versus 79% for EBRT (*p* < 0.05, despite variability in techniques and patient characteristics. Early toxicities of HDR-BT can include erythema, edema, rash, pruritus, and desquamation, while late effects can include skin atrophy, pigmentation changes, alopecia, telangiectasia, fibrosis, and, rarely, ulceration [96]. An overview of select clinical studies assessing outcomes following HDR brachytherapy is presented in Table 3.

### 9.2. Pulse-Dose-Rate Brachytherapy

PDR-BT combines the radiobiological advantages of LDR-BT with the logistical flexibility of HDR-BT delivery [90]. Administered in periodic pulses (e.g., hourly) using a Ir-192 source, PDR-BT allows for optimization of tumor control while reducing normal tissue toxicity by enabling sublethal damage repair between pulses [90]. Treatment typically spans one to several days and requires a specially shielded treatment room [90]. Although less commonly used in skin cancer, PDR-BT may be preferred for lesions needing prolonged irradiation with improved normal tissue tolerance or in settings where HDR-BT infrastructure exists, but LDR-BT dosimetry is desired [90]. Its applications include treatment of perineum, scalp, or extensive cutaneous fields. However, there is a paucity of clinical studies that evaluate PDR-BT outcomes in skin cancer. One recent retrospective study [102] of 155 patients with facial NMSCs treated with HDR-BT or PDR-BT reported excellent 2-year LC (93.9%) and no grade ≥3 late toxicity, although this study highlights the lack of homogenous PDR-BT-specific data.

### 9.3. Low-Dose-Rate Brachytherapy

LDR-BT delivers continuous low-dose radiation (0.4–2 Gy/h) using interstitial implants such as Ir-192 wires, I-125 seeds, or Pd-103 seeds. Historically, it was applied for well-defined superficial lesions or surgical beds when HDR-BT was not available. However, its use has markedly declined due to radiation safety concerns, inpatient requirements, and limited flexibility in dose shaping compared with HDR-BT or IMRT. Today, LDR-BT is primarily of historical relevance, as modern practice favors HDR-BT for its precision, safety, and practicality.

### 9.4. Electronic Brachytherapy

eBT is a modern technology that employs miniaturized X-ray tubes generating low-energy photons (typically around 50 kVp) directly at the treatment site, eliminating the need for radioactive isotopes [103]. Commercial systems such as Esteya^®^* (Elekta AB–Nucletron, Stockholm, Sweden, [104]), Xoft^®^* (Elekta Xoft, Nashua, NH, USA, [105]), and INTRABEAM^®^* (Carl Zeiss Meditec AG, Jena, Germany, [106]) facilitate portable, shielded treatment delivery in outpatient or community dermatology settings. Dosimetrically, eBT achieves a steep dose gradient with rapid fall-off, confining irradiation primarily to superficial lesions ≤5 mm thick while sparing underlying healthy tissue [107]. This permits precise targeting of cosmetically and functionally sensitive anatomical locations [107]. Although eBT mimics the superficial dose distribution of HDR-BT using Ir-192 sources, it lacks the source flexibility and depth control inherent to isotope-based systems [90]. However, the avoidance of complex infrastructure and shielding requirements for eBT makes it particularly appealing for use in dermatology practices and smaller treatment centers. Bhatnagar [108], in one of the earliest studies, analyzed 122 patients with 171 NMSC lesions treated with eBT using the Xoft^®^ system*. Patients received 40 Gy in 8 fractions delivered twice weekly. At a mean follow-up of 10 months (range: 1–28 months), there were no recurrences, and no grade 3 or higher adverse events observed. The system utilizes a miniaturized X-ray source at its tip, capable of delivering HDR-BT, low-energy radiation without radioactive isotopes. Garcia-Martinez et al. [109] evaluated the Esteya^®^ eBT System*, which demonstrated excellent flatness and penumbra, similar to the Valencia applicator, but with an improved percentage depth dose (PDD) that enables treatment of lesions up to 5 mm deep and significantly reduces treatment times due to an increased dose rate. The Esteya^®^ system* comprises a treatment unit with surface applicators, planning software, and a control panel, and its low-energy beam allows treatment within a minimally shielded environment [109]. Despite promising clinical efficacy and logistical advantages, eBT is not widely recommended as a standard treatment modality in major clinical guidelines, potentially due to the lack of large prospective randomized trials and limited long-term data. Furthermore, its application is constrained to superficial lesions, and it is currently not recommended for aggressive or deeply invasive tumors. Nevertheless, eBT is gaining traction as an effective, well-tolerated alternative for patients with contraindications for surgery or those prioritizing cosmesis. Table 4 provides a summary of various studies evaluating eBT outcomes.

* Commercial names are provided for clarity without endorsement—we have no conflicting interest and do not intend to endorse or advertise any particular product.

## 10. Proton Beam Therapy

PBT is an effective modality for treating cutaneous malignancies, particularly in functionally critical regions [115]. It is also becoming more widespread in the setting of re-irradiation, where sparing previously irradiated normal tissues is crucial [115]. The key dosimetric benefit of protons lie in their physical property of the Bragg peak, which delivers the majority of their energy at a defined tissue depth, followed by a rapid fall-off in dose beyond the target [116], thereby substantially reducing unnecessary radiation exposure to adjacent OARs compared to conventional photon techniques [116]. Unlike other EBRT approaches, PBT typically does not require surface bolus or internal shielding, providing a practical and highly precise approach for lesions situated adjacent to critical structures, such as head and neck cutaneous malignancies.

Despite its theoretical and dosimetric advantages, there are currently limited data assessing the benefits of PBT in cutaneous malignancies, with most data derived from small, retrospective single-institution series or small multi-center registry studies with limited long-term follow-up. One study retrospectively reviewed 26 NMSC patients with clinical PNI treated with PBT [117]. Most received standard fractionation of 1.8–2 GyRBE per fraction to 70 GyRBE, while those with tumors adjacent to the optic structures received hyperfractionated regimens of 1.2 GyRBE twice daily to 72–74.4 GyRBE. This approach draws on data suggesting that hyperfractionation with skull base tumors reduced rates of optic neuropathy and auditory damage. At a median follow-up of 2.8 years, the LC rate was 80%, but late grade ≥3 toxicities were seen in 15% of patients, including keratitis and brain necrosis. These complication rates, while comparable to or potentially better than photon-based regimens in similarly complex cases, highlight the ongoing challenge of balancing tumor control with toxicity in the periorbital region. Similarly, PBT was also shown in a multi-center study to achieve durable LC with limited toxicity in head and neck melanomas in the definitive setting [118].

Although PBT offers a compelling therapeutic profile, its use is limited by cost, geospatial availability, and the need for highly specialized planning and delivery infrastructure. Moreover, the risk of severe late toxicities [119], especially when treating near neurovascular structures, warrants caution. Given the paucity of large, homogeneous cohorts and the scarcity of randomized data, further prospective, multi-institutional research with standardized outcome and toxicity reporting is needed to fully define its role in the context of cutaneous malignancies.

## 11. Cost-Effectiveness and Global Availability of Radiotherapy Modalities

Superficial and orthovoltage X-ray therapies remain widely utilized and cost-effective options for managing cutaneous malignancies. Treatment costs vary by modality and fractionation schedule. From one study [120], SXRT ranges from approximately $465 for 5 fractions to $636 for 12 fractions, whereas orthovoltage therapy for a 20-fraction course costs around $3311. EBRT modalities, such as electron beam therapy, are technically more demanding, requiring advanced treatment planning, specialized equipment, and longer delivery times. While EBRT is more complex per fraction, typical treatment courses are shorter—8–10 fractions—resulting in overall costs ranging from $1954 to $2343 [120], comparable to or lower than multi-fraction orthovoltage regimens.

Geospatial availability of these modalities varies. As of 2015, more than 1000 HDR-BT units existed globally, including nearly 400 in low- and middle-income countries [121]; however, the precise number of HDR-BT centers—defined as facilities offering HDR-BT services—may differ, as some centers operate multiple units or share resources, and not all units are necessarily used for skin cancer, since treatment requires custom applicators, imaging guidance, and strict quality assurance protocols. Nonetheless, efforts are being made to expand the availability of HDR-BT units, especially in resource-limited settings. eBT has garnered traction over time and now has over 400 systems worldwide [122]. According to a 2024 market research report by Custom Market Insights, the global eBT market is projected to grow at a compound annual growth rate of 9.48% from 2024 to 2033, with the market size expected to reach USD 947.70 million by 2033 [122]. PBT remains constrained by infrastructure and cost, with roughly 120 operational centers worldwide (49 in the United States, and 19 and 6 in Japan and Germany, respectively) and nearly 70 centers under construction or planned [123]. Consequently, PBT is largely available in high-income, industrialized countries, with limited access in low- and middle-income regions due to the substantial financial and technical requirements of establishing and maintaining these facilities. Even in countries with operational centers, patient access can be further restricted by insurance coverage limitations or denials, particularly for indications where the clinical benefit over conventional RT is still under evaluation. Overall, HDR-BT, electron beam therapy, SXRT, and orthovoltage X-ray therapy continue to represent the most accessible RT options for patients with cutaneous malignancies, with SXRT representing the most cost-effective modality [120].

## 12. Comparative Overview of RT Modalities

Having discussed each modality in detail, it is helpful to provide a concise comparative overview. The following table (Table 5) summarizes the relative strengths and limitations of commonly employed RT techniques for cutaneous malignancies across key clinical dimensions such as tumor depth suitability, cost, availability, logistical considerations, typical indications, toxicity, and cosmetic outcomes. This synthesis provides a practical framework to support clinical decision-making.

## 13. Integrating Systemic Therapy, Immunotherapy and Radiotherapy in Cutaneous Malignancies

Combining systemic agents (targeted therapy, chemotherapy) and ICIs has redefined care for MM and cSCC, and RT is increasingly used as a rational partner in both curative and palliative settings. Neoadjuvant PD-1/CTLA-4 strategies produce substantial gains in resectable MM (SWOG S1801; NADINA), establishing pathological response as a useful surrogate and supporting response-adapted perioperative algorithms [124]. For BRAF-mutant disease, sequencing or early integration of targeted therapy with immunotherapy can improve durability of benefit for selected patients, and triplet (BRAF/MEK + PD-1) approaches remain under active study. In advanced cSCC, PD-1/PD-L1 agents are active and combination biologic strategies (for example avelumab + cetuximab in Alliance A091802) have shown improved progression-free survival (PFS) versus ICI alone, supporting further confirmatory work [125]. Ongoing trials continue to explore optimal sequencing, combination regimens, and biomarkers to guide patient selection, including pivotal studies such as IMspire150 and the KEYNOTE series for melanoma.

RT synergises with immunotherapy through immunogenic cell death, antigen release and microenvironment modulation, and can be deployed to 1. improve LC or palliate symptoms, 2. act as an in situ vaccine to prime systemic immunity (enhancing abscopal responses), and 3. radiosensitise tumors to targeted/immune agents. Multiple clinical series and reviews report improved responses when RT is given concurrently or sequentially with ICIs in melanoma and squamous histologies, and prior RT has been associated with better outcomes for cemiplimab-treated cSCC in retrospective analyses [126]. Practically, integrated care pathways should specify intent (curative vs. palliative), timing (concurrent vs. sequential; neoadjuvant/perioperative), and expected toxicities (immune-related adverse events compounded by RT, possible wound-healing or surgical delay), and where possible record pathologic response, PFS and surgical timing in trial endpoints. Overall, the integration of systemic therapy with RT represents a rapidly evolving paradigm in multidisciplinary skin cancer care, offering the potential for more durable responses and personalized treatment strategies.

## 14. Future Directions

Emerging modalities such as BNCT, fast-neutron therapy, carbon-ion RT, FLASH therapy, rhenium-SCT®* (OncoBeta GmbH, Freiburg, Germany, [127]), MRI-guided RT and artificial intelligence (AI) represent important future directions in the management of skin cancers. BNCT, which exploits preferential uptake of boronophenylalanine via the LAT-1 transporter highly expressed in melanoma cells [128], has shown encouraging results in early-stage MM and angiosarcoma [129], particularly in surgically inoperable cases, with accelerator-based neutron sources [130] broadening clinical feasibility; nevertheless, robust prospective trials, standardized imaging with ^18^F-BPA PET, and refined dosimetry are required before widespread adoption. Other high-linear energy transfer (LET) approaches, including fast-neutron and carbon-ion RT, hold promise for radioresistant cutaneous tumors [131,132,133], offering superior cell kill compared to photons, though clinical data remain limited and toxicity profiles demand careful evaluation, with ongoing work focused on optimizing delivery and exploring synergy with immunotherapy. In parallel, AI is transforming skin cancer RT by optimizing tumor and OAR segmentation, expediting treatment planning, and supporting quality control, image-guided RT, and tumor monitoring [134]. Knowledge-based and deep learning approaches can produce plans comparable to human-generated ones. Beyond RT, AI enhances diagnosis and post-treatment monitoring, achieving dermatologist-level accuracy [135] with real-world deployment in FDA-cleared devices [136] and NHS pilot programs [137]. Additionally, prognostic evaluation through radiomics offers opportunities to standardize treatment, reduce inter-observer variability, and improve dose adequacy, enabling more precise and efficient skin cancer management [138]. While these advances demonstrate the potential for precision, accessibility, and improved outcomes, translation into standard practice will require rigorous clinical validation, integration into workflows, and safeguards for equity, data privacy, and clinician-AI collaboration.

* Commercial names are provided for clarity without endorsement—we have no conflicting interest and do not intend to endorse or advertise any particular product.

## 15. Limitations

This review is limited by the rarity of data on radiotherapeutic modalities in skin cancer management, with studies showing heterogeneity in patient selection, treatment protocols, and outcome reporting. The lack of dosimetric standardization across modalities makes direct technical comparisons difficult. Important factors such as RT modality, RT doses, cosmetic outcomes, QoL measures, and toxicity grading, have been inconsistently reported or absent. Some studies are from older treatment eras, limiting applicability to modern practice with advanced imaging and planning techniques. Selection bias may exist as such cohorts often include patients unsuitable for surgery, and rarely include immunocompromised populations. Additionally, anatomical subsites were not uniformly stratified. There is also a notable lack of high-quality studies evaluating RT specifically for MM and rarer tumors, further limiting evidence-based recommendations for this subgroup. More robust research is required to accurately evaluate the cost-effectiveness of different RT modalities and to document their availability and utilization across diverse healthcare settings globally, particularly in low-resource environments where access to advanced technologies may be challenging. These limitations highlight the need for more standardized, prospective research to better define optimal radiation strategies in skin cancer.

## 16. Conclusions

As the therapeutic landscape for cutaneous malignancies continues to evolve, RT remains a key pillar in both the definitive and adjuvant settings, offering a non-invasive alternative with excellent oncologic and cosmetic outcomes. The rise in novel modalities such as eBT reflects an increasing emphasis on precision, convenience, and outpatient feasibility that is especially relevant in aging populations and those with comorbidities. Yet, the field of RT is challenged by inconsistencies in dose-fractionation regimens, limited homogenous comparative data, and variability in access to advanced RT modalities. Notably, there are few prospective clinical trials and limited robust comparative evidence supporting the use of advanced RT techniques, such as IMRT and PBT, in cutaneous malignancies. Thus, future efforts must focus on generating rigorous data while ensuring that clinical innovation remains aligned with patient needs, practical feasibility and overall benefit.

## Figures and Tables

**Table 1:** Evidence-Based Strategies for Cutaneous Malignancies by Tumor Type and Treatment Intent.

Histology	Risk/Size/Stage	Primary (Preferred Treatment)	Primary RT (Definitive)	Adjuvant RT	Palliative RT	Nodal Management	Immunotherapy/Systemic Therapy	Considerations: Immunocompromised Individuals and Special Cases
cBCC	Low-risk: ≤2 cm, superficial. High-risk: >2 cm, infiltrative, periorificial.	Surgery/Mohs	Patient/clinician preference or if surgery contraindicated. Techniques: superficial/orthovoltage, electrons, HDR-BT (surface applicators/molds), eBT, conformal photons for deeper lesions. PBT can be considered for periorbital or skull base involvement.	Used for positive margins or PNI.	Symptom control	Nodal mets very rare; routine nodal RT not indicated.	Hedgehog inhibitors (vismodegib/sonidegib) or PD-1 inhibitors for advanced/metastatic disease.	Immunocompromised: consider lower threshold for definitive/adjuvant RT due to aggressive growth, higher recurrence risk
cSCC	Low-risk: ≤2 cm, ≤6 mm depth, well/mod diff, no PNI. High-risk: >2 cm, ≥6 mm depth, poor-diff, PNI, recurrent, immunosuppressed.	Wide excision ± Mohs; LN evaluation if clinically suspicious.	Definitive RT for inoperable lesions or patient/clinician preference. Techniques: superficial/orthovoltage or electrons for small superficial lesions; IMRT/VMAT/3D-CRT for deep/PNI; HDR-BT/eBT for small superficial (<4–5 mm, ≤2–3 cm) lesions; PBT can be considered for periocular/skull base tumors.	High-risk features: positive margins, extensive PNI, large primary; elective nodal RT as indicated; treat along nerve to skull base if PNI.	Symptomatic advanced disease with electrons/photons.	Elective nodal RT may be considered in high-risk primary sites (e.g., lip, ear, deep/large tumors, poorly differentiated, immunosuppressed) -especially in head/neck locations where surgical nodal management may be limited and risk ≥15%. Clinically involved nodes and named-nerve PNI should receive therapeutic nodal or nerve tract RT	PD-1 inhibitors (cemiplimab, pembrolizumab) for locally advanced/metastatic disease.	Immunocompromised: higher recurrence/metastasis; consider elective nodal RT more liberally; lower threshold for definitive RT
Melanoma—LM (in situ)	Extensive facial LM, elderly/comorbid	Wide local excision	RT acceptable if surgery not feasible or disfiguring; patient/clinician preference. Techniques: superficial/orthovoltage (~5 mm depth), HDR-BT/eBT for small convex sites. PBT rarely indicated for periocular lesions.		Not indicated	Not indicated	Not indicated	Immunocompromised: may have accelerated progression; careful RT planning, close follow-up
Melanoma—invasive/desmoplastic	Breslow depth guides staging; desmoplastic/neurotropic high-risk	Wide local excision ± SLNB	Adjuvant RT more common; primary RT rare, consider HDR-BT/eBT for small superficial lesions if surgery not feasible. PBT may be considered in head/neck sites near OARs.		Palliative for metastases: SRS, hypofractionated RT.	Treat nodal disease per melanoma nodal guidelines; consider RT if unresectable or extracapsular extension.	ICIs/targeted therapy for advanced disease.	Satellite/in-transit mets: RT can be used for LC, symptom relief; electrons, IMRT, or SXRT depending on size/number/location
MCC	Small primary lesions common; high regional spread risk	Wide excision + SLNB	Definitive RT for inoperable cases. Techniques: electrons/photons standard; HDR-BT/eBT occasionally for superficial lesions; protons for periocular/complex head & neck sites.		Palliative RT for symptomatic locoregional or distant disease (short courses).	SLNB strongly recommended; nodal RT ± dissection if positive; elective nodal RT often used.	PD-L1/PD-1 inhibitors (avelumab, pembrolizumab, nivolumab) for advanced/metastatic disease.	Immunocompromised: higher recurrence risk; may benefit from wider RT fields or elective nodal RT
Other rare cutaneous malignancies	Cutaneous lymphomas, adnexal tumors (sebaceous, eccrine poro/hidradenocarcinoma)	Surgery when possible; lymphomas treated medically	RT frequently used: superficial/electrons for localized lymphoma; TSEBT for generalized disease; HDR/eBT for small superficial adnexal tumors. PBT in selected complex head/neck/adnexal lesions.		Hypofractionated short courses for symptom control.	Lymphoma nodal management stage-dependent; adnexal tumors treated like high-risk head/neck skin cancers.	Lymphoma systemic therapy per hematology protocols; adnexal tumors occasionally receive chemo/targeted therapy; limited evidence.	Immunocompromised: consider early RT for aggressive disease; close monitoring for recurrence

Abbreviations: PD-1 inhibitor, programmed death-1 inhibitor; HDR-BT, high-dose-rate brachytherapy; eBT, electronic brachytherapy; PBT, proton beam therapy; RT, radiotherapy; PNI, perineural invasion; LN evaluation, lymph node evaluation; IMRT, intensity-modulated radiation therapy; VMAT, volumetric modulated arc therapy; 3D-CRT, three-dimensional conformal radiotherapy; LM, lentigo maligna; SRS, stereotactic radiosurgery; SLNB; sentinel lymph node biopsy; ICIs, immune-checkpoint inhibitors; OARs, organs at risk; LC, local control; SXRT, superficial X-ray therapy; PD-L1 inhibitor, programmed death-ligand 1 inhibitor; TSEBT, total skin electron beam therapy.

**Table 2:** Overview of Select Clinical Studies and Analyses Evaluating Kilovoltage X-ray Therapy outcomes.

Study (Author, Year)	Histology and Disease Site	No. of Patients, No. of Lesions	RT Modality	Dose and Fractionation	Length of Follow-up	LC and/or Recurrence Rates	Cosmesis and Toxicity
Mattia et al., 2024 [52]	Nodular cBCC, superficial cBCC, invasive cSCC, cSCC in situ, and combined histology	1082 patients, 2490 lesions	SXRT	Average dose 35.7 Gy over 5.47 fractions	Up to 22 years, minimum 1 month	All subtypes recurrence: 2-year: 2.2% 5-year: 6.0% 10-year: 10.5% cBCC recurrence: 2-year: 2.8% 5-year: 6.9% 10-year: 12.4% cSCC recurrence: 2-year: 2.0% 5-year: 5.8% 10-year: 9.9%	Not specified
Green et al., 2023 [53]	cBCC and cSCC	891 patients, 2179 lesions	SXRT	Not specified (full study inaccessible)	Up to 10 years	cBCC Recurrence: 2-year: 2.8% 5-year: 7.2% 10-year: 9.6% cSCC Recurrence: 2-year: 2.2% 5-year: 6.5% 10-year: 8.9%	Not specified (full study inaccessible)
Duinkerken et al., 2016 [54]	Head & neck favorable cBCC	232 patients, 253 lesions	Orthovoltage X-ray RT	2 regimens: Non-periocular lesions, very old patients with poor overall health: 4.5 Gy in 10 fractions All other lesions: 3 Gy in 18 fractions	Maximum 5 years (range 1 month–5 years)	1-year LC 98.9%, 3-year LC 97.5%, 5-year LC 96.3%	Acute toxicity: self-resolving Late toxicity: not significant Excellent cosmesis and no functional impairments
Krema et al., 2013 [55]	Medial canthal cBCC	90 patients	Orthovoltage X-ray RT	Median dose was 35 Gy delivered in 5 daily fractions, used in 59 (66%) of patients, with 16 (18%) treated with 45 Gy in 10 daily fractions and 9 (10%) with 50 Gy in 20 daily fractions.	Median 80 months	10-year LC: 94%	Toxicity: eyelash loss occurred in 59% of patients, epiphora occurred in 51% of patients, dry eye occurred in 14% of patients, conjunctival scarring occurred in 11% of patients. No corneal complications.
Marconi et al., 2013 [48]	cBCC	597 patients, 1021 lesions	Orthovoltage X-ray RT	3 regimens: 2.5 Gy in 22 fractions (55 Gy total); 2.5 Gy in 20 fractions (50 Gy total); 2.0 Gy in 30 fractions (60 Gy total)	Median 44 months (range 1–406 months)	All subtypes LC: 5-year 95%, 10-year 92.9% cBCC LC: 5-year 95.6%, 10-year 94.3% cSCC LC: 5-year 91.9%, 10-year 87.3%	Toxicity: 8.88% of lesions developed grade 3+ acute toxicity; no significant difference in toxicity by fractionation.
Zagrodnik et al., 2003 [56]	Nodular cBCC, superficial cBCC, sclerosing cBCC	148 patients, 175 lesions	SXRT	3 regimens based on lesion size (cm): <2 cm: 8 Gy in 5–6 fractions (40–48 Gy total) 2–5 cm: 4 Gy in 10–13 fractions (40–52 Gy total) >5 cm: 2 Gy in 26–30 fractions (52–60 Gy total)	Median 48 months	Overall 5-year recurrence rate: 15.8% By subtype: nodular: 8.2% recurrence, superficial: 26.1% recurrence, sclerosing: 27.7% recurrence Higher recurrence rate associated with sclerosing subtype, and p53 and Bcl-2 expression; lower recurrence rate associated with nodular subtype.	Not specified

Abbreviations: RT, radiotherapy; cBCC, cutaneous basal cell carcinoma; cSCC, cutaneous squamous cell carcinoma; SXRT, superficial X-ray therapy; LC, local control.

**Table 3:** Overview of Select Clinical Studies Evaluating HDR-BT outcomes.

Study (Author, Year)	Histology	No. of Patients, No. of Lesions	Dose and Fractionation	Applicators or Delivery Method	Length of Follow-up	LC and/or Recurrence Rates	Cosmesis and Toxicity
Cirulia et al., 2025 [97]	cBCC and cSCC	39 patients, 46 lesions	2 regimens: 40 Gy in 4 fractions or 30 Gy in 3 fractions	Leipzig applicator	Mean 25.1 months (range 1 month–77 months)	100% LC and 100% disease-specific survival at 2-year follow-up.	Acute toxicity: grade 1 in 39.1% of patients, grade 2 in 10.9% of patients, grade 3 in 15.2% of patients. No acute toxicity was observed in 34.8% of patients. Late toxicity: not detected in 76.1% of patients.
Monge-Cadet et al., 2024 [98]	Facial cBCC and cSCC	67 patients, 67 lesions	40 Gy in 8 fractions delivered over 5 consecutive days	Flexible interstitial implant tubes	Median 28 months and 3 years	After median follow-up of 28 months, 8 patients developed local recurrence, 3 developed nodal recurrence, and 3 developed metastatic recurrence. 87.05% LC at 3 years for all patients.	Acute toxicity: All patients experienced grade 1 and grade 2 acute side-effects, 1 patient experienced grade 3 acute side effects. Late toxicity: no patients with severe late toxicity. Of the patients who reported QoL outcomes, 77.8% recommended the treatment.
Oliviera et al., 2024 [99]	Lower eyelid cBCC	58 patients, 58 lesions	36–40 Gy in 9–10 fractions, twice daily over 5 days.	Interstitial catheters	Median 44 months	95% and 100% LC in adjuvant and radical groups, respectively. 4 local relapses.	Acute toxicity: 76% of patients developed acute toxicity (1 grade 3 dermatitis) Late toxicity: 56% of patients. Excellent/very good cosmesis in 93%.
Bilski et al., 2022 [100]	Peri-auricular NMSC	33 patients	7 Gy per fraction, time intervals from 6 h (interstitial) up to 7 days (contact); total dose range 7–49 Gy	Contact HDR-BT and interstitial HDR-BT	Mean 29.75 months (range 2–64 months)	97% LC with 1 local recurrence (3%)	Toxicity: no toxicity in 15 (45.5%) patients, grade 1 toxicity in 15 (45.5%) patients, grade 2 toxicity in 2 (6%) patients, no 3 or higher toxicity.
Renard et al., 2021 [101]	Facial NMSC	66 patients, 71 lesions	2 regimens: 7 Gy day 1 then 8 Gy in 4 fractions over next 4 days or 4 Gy over 5 days (post-operative cases)	Interstitial catheters	Median 15.3 months	98.5% complete response at median 15.3 month follow-up. 3% local recurrence at median 20.5 month follow-up	Acute toxicity: grade 3 acute dermatitis in 4 (6.1%) patients and grade 3 mucositis in 3 (4.5%) patients. These resolved within 3 months. Late toxicity: late hypopigmentation in 4 patients.

Abbreviations: cBCC, cutaneous basal cell carcinoma; cSCC, cutaneous squamous cell carcinoma; NMSC, non-melanoma skin cancer; LC, local control; QoL, quality of life; RTOG, Radiation Therapy Oncology Group.

**Table 4:** Overview of Select Clinical Studies Evaluating eBT outcomes.

Study (Author, Year)	Histology and Disease Site	No. of Patients, No. of Lesions	Dose and Fractionation	Length of Follow-Up	LC and/or Recurrence Rates	Cosmesis and Toxicity
Cheng et al., 2024 [110]	cBCC and cSCC	205 patients, 236 lesions	69–72 GyBED (various fraction regimes)	Median 24.2 months (maximum 73.5 months)	99.6% LC (1 recurrence)	Acute toxicity: erythema (34.1% at 1 month), resolved within 6 months Late toxicity: Hypopigmentation, telangiectasia Cosmesis: HCP-rated E/G at 36 months: 83.6%; Patient-rated E/G at 36 months: 86.6%
Dogett et al., 2023 [111]	cBCC and cSCC	183 patients, 185 lesions	40 Gy in 8 fractions (given twice weekly)	Median 7.5 years (range 5–9.5 years)	98.9% LC	Toxicity: grade 1 hypopigmentation (65.9% of patients), telangiectasia (22.2% of patients), rare grade 1-2 scarring (1.1% of patients), hyperpigmentation (1.1% of patients, induration (0.5% of patients)
Goyal et al., 2021 [112]	cBCC and cSCC	33 patients, 50 lesions	Median BED: 50 Gy total dose to a 0.1–0.5 cm depth (various fractionation regimens)	Mean 45.6 months	97% LC	Acute toxicity: grade 3 toxicity occurred in 9 lesions (18%), grade 4 toxicity occurred in 4 lesions (8%) Late toxicity: none reported at median 45.6 month follow-up
Ballister-Sánchez et al., 2015 [113]	cBCC	20 patients, 23 lesions	42 Gy in 6 fractions (given twice weekly)	6 months for all cases	100% LC	Toxicity: Mild erythema post-4th fraction; no serious toxicity reported Excellent cosmesis in >60%; subtle changes in remaining
Paravati et al., 2015 [114]	cBCC	127 patients, 154 lesions	40 Gy in 8 fractions	Median 16.1 months (range 3.4–34.8 months)	98.7% LC	Acute toxicity: grade 0–1 (52.6%), grade 2 (34.4%), grade 3 (13%) Late toxicity: grade 0–1 (94.2%), grade 2 (5.8%) Excellent cosmesis in 94.2%, good outcomes in 3.3% and fair/poor in 1.4%

Abbreviations: cBCC, cutaneous basal cell carcinoma; cSCC, cutaneous squamous cell carcinoma; LC, local control; HCP-rated E/G, healthcare professional-rated Excellent/Good; patient-rated E/G, patient-rated Excellent/Good.

**Table 5:** Summary comparison of RT modalities in cutaneous malignancies.

Modality	Tumor Depth Suitability	Cost	Availability	Logistical Benefits	Technical Limitations	Typical Indications	Toxicity	Cosmesis
Orthovoltage/superficial X-rays	Very superficial (<5 mm)	Low	Widely available (declining use in high-income settings)	Simple setup, outpatient treatment	Limited depth penetration	Small, superficial lesions; palliative lesions; selected adjuvant cases for superficial margins	Skin atrophy, telangiectasia	Overall good
3D-CRT	Up to several cm	Moderate	Widely available	Relatively straightforward planning	Less conformal than IMRT/VMAT	Larger or deeper cutaneous tumors; post-op adjuvant treatment; nodal irradiation in select cases	Moderate dose to adjacent tissues	Variable; depends on field size
IMRT	Deep or complex volumes	High	Widely available in developed centers	Highly conformal, spares normal tissue	Longer planning and delivery times	Irregular target volumes; head & neck skin cancers; post-op adjuvant; nodal irradiation; re-irradiation	Lower normal tissue dose, but risk of low-dose bath	Good, especially in cosmesis-sensitive areas
VMAT	Similarly to IMRT, faster delivery	High	Increasingly available	Shorter treatment time than IMRT	Requires advanced planning software	Similarly to IMRT; post-op adjuvant; nodal coverage; complex geometry; re-irradiation	Similarly to IMRT	Comparable to IMRT
Electron beam therapy	Up to ~5 cm, sharp distal fall-off	Moderate	Widely available	Rapid delivery, predictable dose fall-off	Limited to uniform fields; complex shapes require multiple energies	Superficial to moderately deep tumors; post-op scar boost; skin lymphomas; adjuvant or definitive treatment of localized lesions; re-irradiation of previously treated superficial sites	Erythema, desquamation	Excellent for appropriately selected depths
HDR-BT	Superficial (3–10 mm)	Moderate-high	Available at specialized centers	Outpatient or short-course treatment	Requires specialized applicators and expertise	Small, well-defined lesions; cosmesis-critical sites (face, ears, nose); adjuvant treatment for positive or close margins; boost therapy; re-irradiation	Ulceration, hypopigmentation	Excellent (esp. face, nose, ears)
PDR-BT	Similarly to HDR-BT (fractionated, protracted)	Moderate-high	Less widely available	Fractionated delivery may reduce toxicity	Requires specialized equipment	Alternative to HDR-BT in select centers; adjuvant therapy; boost to surgical bed; palliation of superficial lesions; re-irradiation	Similarly to HDR-BT, potentially reduced late effects	Excellent
eBT	Very superficial (≤5 mm)	Moderate	Limited availability (office-based units)	Portable, office-based	Limited depth penetration	Outpatient settings; boost to surgical margins; superficial palliation; re-irradiation	Similarly to HDR-BT, low acute toxicity	Very good
PBT	Deep, complex, sparing OARs	High	Limited to major centers	Excellent normal tissue sparing	Very high cost, limited access	Data emerging for definitive treatment of skin lesions; deep or recurrent lesions; periorbital and scalp tumors; adjuvant therapy in high-risk cases; nodal coverage in select patients; re-irradiation	Reduced integral dose	Good especially when OAR sparing is critical

Abbreviations: 3D-CRT, three-dimensional conformal radiotherapy; IMRT, intensity-modulated radiation therapy; VMAT, volumetric modulated arc therapy; HDR-BT, high-dose-rate brachytherapy; PDR-BT, pulse-dose-rate brachytherapy; eBT, electronic brachytherapy; PBT, proton beam therapy; OARs, organs at risk.

## Data Availability

No new data were created or analyzed in this study.

## References

[1] International Agency for Research on Cancer (2025). Skin Cancer. https://www.iarc.who.int/cancer-type/skin-cancer/.

[2] Bray F., Laversanne M., Sung H., Ferlay J., Siegel R.L., Soerjomataram I., Jemal A. (2024). Global cancer statistics 2022: GLOBOCAN estimates of incidence and mortality worldwide for 36 cancers in 185 countries. CA Cancer J. Clin..

[3] Wang M., Gao X., Zhang L. (2025). Recent global patterns in skin cancer incidence, mortality, and prevalence. Chin. Med. J..

[4] Hu W., Fang L., Ni R., Zhang H., Pan G. (2022). Changing trends in the disease burden of non-melanoma skin cancer globally from 1990 to 2019 and its predicted level in 25 years. BMC Cancer.

[5] Apalla Z., Nashan D., Weller R.B., Castellsagué X. (2017). Skin cancer: Epidemiology, disease burden, pathophysiology, diagnosis, and therapeutic approaches. Dermatol. Ther..

[6] Kim J.Y., Dao H. (2023). Physiology, Integument. StatPearls [Internet].

[7] National Cancer Institute Cancer Stat Facts: Melanoma of the Skin. SEER Cancer Statistics. https://seer.cancer.gov/statfacts/html/melan.html.

[8] Damsky W.E., Rosenbaum L.E., Bosenberg M. (2010). Decoding melanoma metastasis. Cancers.

[9] American Cancer Society Key Statistics for Melanoma Skin Cancer. https://www.cancer.org/cancer/melanoma-skin-cancer/about/key-statistics.html.

[10] Aggarwal P., Knabel P., Fleischer A.B. (2021). United States burden of melanoma and non-melanoma skin cancer from 1990 to 2019. J. Am. Acad. Dermatol..

[11] Hadian Y., Howell J.Y., Ramsey M.L., Buckley C. (2024). Cutaneous Squamous Cell Carcinoma. StatPearls.

[12] McDaniel B., Steele R.B. (2025). Basal Cell Carcinoma. StatPearls [Internet].

[13] Brady M., Puckett Y. (2025). Merkel Cell Carcinoma of the Skin. StatPearls [Internet].

[14] Roth J.J., Granick M.S. (1997). Squamous Cell and Adnexal Carcinomas of the Skin. Clin. Plast. Surg..

[15] Prickett K.A., Ramsey M.L. (2023). Mohs micrographic surgery. StatPearls [Internet].

[16] Genders R.E., Marsidi N., Michi M., Henny E.P., Goeman J.J., van Kester M.S. (2020). Incomplete excision of cutaneous squamous cell carcinoma; systematic review of the literature. Acta Derm. Venereol..

[17] National Comprehensive Cancer Network (2025). Squamous Cell Skin Cancer, Version 2.2025. NCCN Clinical Practice Guidelines in Oncology.

[18] National Comprehensive Cancer Network (2025). Cutaneous Melanoma, Version 2.2025. NCCN Clinical Practice Guidelines in Oncology.

[19] National Comprehensive Cancer Network (2025). Non-Melanoma Skin Cancer, Version 2.2025. NCCN Clinical Practice Guidelines in Oncology.

[20] Van Egmond S., Wakkee M., Hoogenraad M., Korfage I.J., Mureau M.A.M., Lugtenberg M. (2022). Complex skin cancer treatment requiring reconstructive plastic surgery: An interview study on the experiences and needs of patients. Arch. Dermatol. Res..

[21] Grob J.J., Gaudy-Marqueste C., Guminski A., Malvehy J., Basset-Seguin N., Bertrand B., Fernandez-Peñas P., Kaufmann R., Zalaudek I., Fargnoli M.C. (2021). Position statement on classification of basal cell carcinomas. Part 2: EADO proposal for new operational staging system adapted to basal cell carcinomas. J. Eur. Acad. Dermatol. Venereol..

[22] Stratigos A.J., Garbe C., Dessinioti C., Lebbe C., van Akkooi A., Bataille V., Bastholt L., Dreno B., Dummer R., Fargnoli M.C. (2023). European consensus-based interdisciplinary guideline for invasive cutaneous squamous cell carcinoma. Part 1: Diagnostics and prevention—Update 2023. Eur. J. Cancer.

[23] Ferini G., Molino L., Bottalico L., De Lucia P., Garofalo F. (2021). A small case series about safety and effectiveness of a hypofractionated electron beam radiotherapy schedule in five fractions for facial non-melanoma skin cancer among frail and elderly patients. Rep. Pr. Oncol. Radiother..

[24] Yosefof E., Kurman N., Yaniv D. (2023). The role of radiation therapy in the treatment of non-melanoma skin cancer. Cancers.

[25] National Comprehensive Cancer Network (2025). Merkel Cell Carcinoma, Version 2.2025. NCCN Clinical Practice Guidelines in Oncology.

[26] National Comprehensive Cancer Network (2025). Primary Cutaneous Lymphomas, Version 3.2025. NCCN Clinical Practice Guidelines in Oncology.

[27] Likhacheva A., Awan M., Barker C.A., Bhatnagar A., Bradfield L., Brady M.S., Buzurovic I., Geiger J.L., Parvathaneni U., Zaky S. (2020). Definitive and postoperative radiation therapy for basal and squamous cell cancers of the skin: Executive summary of an American Society for Radiation Oncology clinical practice guideline. Pract. Radia.t Oncol..

[28] Guinot J.L., Rembielak A., Perez-Calatayud J., Rodríguez-Villalba S., Skowronek J., Tagliaferri L., Guix B., Gonzalez-Perez V., Valentini V., Kovacs G. (2018). GEC-ESTRO ACROP recommendations in skin brachytherapy. Radiother Oncol..

[29] Cox J.D., Stetz J., Pajak T.F. (1995). Toxicity criteria of the Radiation Therapy Oncology Group (RTOG) and the European Organization for Research and Treatment of Cancer (EORTC). Int. J. Radiat. Oncol. Biol. Phys..

[30] U.S. Department of Health and Human Services, National Institutes of Health, National Cancer Institute (2009). Common Terminology Criteria for Adverse Events (CTCAE).

[31] Cognetta A.B., Wolfe C.M., Goldberg D.J., Hong H.G. (2016). Practice and educational gaps in radiation therapy in dermatology. Dermatol. Clin..

[32] Bhupalam V., Hetzel J.D., Awad N., Nestor M.S. (2024). Superficial radiation therapy in dermatology: A historical perspective. Dermatol. Rev..

[33] Safigholi H., Song W.Y., Meigooni A.S. (2015). Optimum radiation source for radiation therapy of skin cancer. J. Appl. Clin. Med. Phys..

[34] Nestor M.S., Berman B., Goldberg D., Cognetta A.B., Gold M., Roth W., Cockerell C.J., Glick B. (2019). Consensus guidelines on the use of superficial radiation therapy for treating nonmelanoma skin cancers and keloids. J. Clin. Aesthet. Dermatol..

[35] Velindre University NHS Trust (2016). Clinical Protocol: Skin Malignancies. Issue 5. https://velindre.nhs.wales/about-us/publications/freedom-of-information/foia-disclosure-logs-2023/january-2023/corp-2023-010-skin-cancer-pathway-management-attachment-2/.

[36] Han H., Gade A., Ceci F.M., Lawson A., Auerbach S., Nestor M.S. (2023). Superficial radiation therapy for nonmelanoma skin cancer: A review. Dermatol. Ther..

[37] Cognetta A.B., Howard B.M., Heaton H.P., Stoddard E.R., Hong H.G., Green W.H. (2012). Superficial X-ray in the treatment of basal and squamous cell carcinomas: A viable option in select patients. J. Am. Acad. Dermatol..

[38] Schulte K.W., Lippold A., Auras C., Müller-Pannes H., Rupprecht R., Suter L. (2005). Soft X-ray therapy for cutaneous basal cell and squamous cell carcinomas. J. Am. Acad. Dermatol..

[39] Lee Y.C., Davis S.D., Romaguera W., Chaswal V., Tolakanahalli R., Gutierrez A.N., Kalman N.S. (2023). Implementation of superficial radiation therapy (SRT) using SRT-100 Vision™ for non-melanoma skin cancer in a Radiation Oncology clinic. J. Appl. Clin. Med. Phys..

[40] Moloney M., Harris P.M., Kaczmarski P., Zheng S., Ladd D., Serure D., Malik A., Yu L. (2025). Updated results of 3,050 non-melanoma skin cancer (NMSC) lesions in 1,725 patients treated with high resolution dermal ultrasound-guided superficial radiotherapy, a multi-institutional study. BMC Cancer.

[41] Tran A., Moloney M., Kaczmarski P., Zheng S., Desai A., Desai T., Yu L. (2023). Analysis of image-guided superficial radiation therapy (IGSRT) on the treatment of early-stage non-melanoma skin cancer (NMSC) in the outpatient dermatology setting. J. Cancer Res. Clin. Oncol..

[42] Wilson L.D., Kacinski B.M., Jones G.W. (1998). Local superficial radiotherapy in the management of minimal stage IA cutaneous T-cell lymphoma (Mycosis Fungoides). Int. J. Radiat. Oncol. Biol. Phys..

[43] Kang S.H., Haydu L.E., Goh R.Y.H., Fogarty G.B. (2012). Radiotherapy is associated with significant improvement in local and regional control in Merkel cell carcinoma. Radiat. Oncol..

[44] Plastic Surgery Key Radiotherapy. https://plasticsurgerykey.com/radiotherapy-2/.

[45] Amdur R.J., Kalbaugh K.J., Ewald L.M., Parsons J.T., Mendenhall W.M., Bova F.J., Million R.R. (1992). Radiation therapy for skin cancer near the eye: Kilovoltage X-rays versus electrons. Int. J. Radiat. Oncol. Biol. Phys..

[46] Bell B.I., Vercellino J., Brodin N.P., Velten C., Nanduri L.S.Y., Nagesh P.K.B., Tanaka K.E., Fang Y., Wang Y., Macedo R. (2022). Orthovoltage X-rays exhibit increased efficacy compared with γ-rays in preclinical irradiation. Cancer Res..

[47] Abbatucci J.S., Boulier N., Laforge T., Lozier J.C. (1989). Radiation therapy of skin carcinomas: Results of a hypofractionated irradiation schedule in 675 cases followed more than 2 years. Radiother. Oncol..

[48] Marconi D., Resende B., Rauber E., Soares P., Fernandes J., Carvalho A., Kupelian P., Chen A. (2014). Head and neck nonmelanoma skin cancer treated by orthovoltage radiation: An analysis of 1021 cases. Int. J. Radiat. Oncol. Biol. Phys..

[49] Hendrickx A., Cozzio A., Plasswilm L., Panje C.M. (2020). Radiotherapy for lentigo maligna and lentigo maligna melanoma—A systematic review. Radiat. Oncol..

[50] Schmid-Wendtner M.H., Brunner B., Konz B., Kaudewitz P., Wendtner C.M., Peter R.U., Plewig G., Volkenandt M. (2000). Fractionated radiotherapy of lentigo maligna and lentigo maligna melanoma in 64 patients. J. Am. Acad. Dermatol..

[51] Kharofa J., Currey A., Wilson J.F. (2013). Patient-reported outcomes in patients with nonmelanomatous skin cancers of the face treated with orthovoltage radiation therapy: A cross-sectional survey. Int. J. Radiat. Oncol. Biol. Phys..

[52] Mattia A., Thompson A., Lee S.K., Hong H.G., Green W.H., Cognetta A.B. (2024). Superficial X-ray in the treatment of nonaggressive basal and squamous cell carcinoma in the elderly: A 22-year retrospective analysis. J. Am. Acad. Dermatol..

[53] Green W.H., Mattia A., Thompson A., Lee S., Hong G., Cognetta A. (2023). Superficial X-ray in the treatment of basal and squamous cell carcinomas for select patients: The second decade. J. Am. Acad. Dermatol..

[54] Duinkerken C.W., Lohuis P.J.F., Heemsbergen W., Zupan-Kajcovski B., Navran A., Hamming-Vrieze O., Klop W.M.C., Balm F.J.M., Al-Mamgani A. (2016). Orthovoltage for basal cell carcinoma of the head and neck: Excellent local control and low toxicity profile. Laryngoscope.

[55] Krema H., Herrmann E., Albert-Green A., Payne D., Laperriere N., Chung C. (2013). Orthovoltage radiotherapy in the management of medial canthal basal cell carcinoma. Br. J. Ophthalmol..

[56] Zagrodnik B., Kempf W., Seifert B., Müller B., Burg G., Urosevic M., Dummer R. (2003). Superficial radiotherapy for patients with basal cell carcinoma: Recurrence rates, histologic subtypes, and expression of p53 and Bcl-2. Cancer.

[57] Verhey L.J. (1999). Comparison of three-dimensional conformal radiation therapy and intensity-modulated radiation therapy systems. Semin. Radiat. Oncol..

[58] Cho B. (2018). Intensity-modulated radiation therapy: A review with a physics perspective. Radiat. Oncol. J..

[59] Mattes M.D., Zhou Y., Berry S.L., Barker C.A. (2016). Dosimetric comparison of axilla and groin radiotherapy techniques for high-risk and locally advanced skin cancer. Radiat. Oncol. J..

[60] Henderson M.A., Burmeister B.H., Ainslie J., Fisher R., Di Iulio J., Smithers B.M., Hong A., Shannon K., Scolyer R.A., Carruthers S. (2015). Adjuvant lymph-node field radiotherapy versus observation only in patients with melanoma at high risk of further lymph-node field relapse after lymphadenectomy (ANZMTG 01.02/TROG 02.01): 6-year follow-up of a phase 3, randomised controlled trial. Lancet Oncol..

[61] Cheung K.Y. (2006). Intensity modulated radiotherapy: Advantages, limitations and future developments. Biomed. Imaging Interv. J..

[62] Hong T.S., Ritter M.A., Tomé W.A., Harari P.M. (2005). Intensity-modulated radiation therapy: Emerging cancer treatment technology. Br. J. Cancer.

[63] Chao K.S.C., Ozyigit G., Tran B.N., Cengiz M., Dempsey J.F., Low D.A. (2003). Patterns of failure in patients receiving definitive and postoperative IMRT for head-and-neck cancer. Int. J. Radiat. Oncol. Biol. Phys..

[64] Dawson L.A., Anzai Y., Marsh L., Martel M.K., Paulino A., Ship J.A., Eisbruch A. (2000). Patterns of local-regional recurrence following parotid-sparing conformal and segmental intensity-modulated radiotherapy for head and neck cancer. Int. J. Radiat. Oncol. Biol. Phys..

[65] Matthiesen C., Thompson J.S., Forest C., Ahmad S., Herman T., Bogardus C. (2011). The role of radiotherapy for T4 non-melanoma skin carcinoma. J. Med. Imaging Radiat. Oncol..

[66] Hallemeier C.L., Garces Y.I., Neben-Wittich M.A., Olivier K.R., Shon W., García J.J., Brown P.D., Foote R.L. (2013). Adjuvant hypofractionated intensity modulated radiation therapy after resection of regional lymph node metastases in patients with cutaneous malignant melanoma of the head and neck. Pract. Radiat. Oncol..

[67] Miéville F.A., Pitteloud N., Achard V., Lamanna G., Pisaturo O., Tercier P.A., Allal A.S. (2023). Post-mastectomy radiotherapy: Impact of bolus thickness and irradiation technique on skin dose. Z. Med. Phys..

[68] Yang R., Xu S., Jiang W., Xie C., Wang J. (2009). Integral dose in three-dimensional conformal radiotherapy, intensity-modulated radiotherapy and helical tomotherapy. Clin. Oncol. (R. Coll. Radiol.).

[69] Unkelbach J., Bortfeld T., Craft D., Alber M., Bangert M., Bokrantz R., Chen D., Li R., Xing L., Men C. (2015). Optimization approaches to volumetric modulated arc therapy planning. Med. Phys..

[70] Afrin K.T., Ahmad S. (2021). Clinical differences among volumetric modulated arc therapy, intensity modulated radiation therapy, and 3D conformal radiation therapy in prostate cancer: A brief review study. Multidiscip. Cancer Investig..

[71] Holt A., Van Gestel D., Arends M.P., Korevaar E.W., Schuring D., Kunze-Busch M.C., Louwe R.J.W., van Vliet-Vroegindeweij C. (2013). Multi-institutional comparison of volumetric modulated arc therapy vs. intensity-modulated radiation therapy for head-and-neck cancer: A planning study. Radiat. Oncol..

[72] Bray F.N., Simmons B.J., Wolfson A.H., Nouri K. (2016). Acute and chronic cutaneous reactions to ionizing radiation therapy. Dermatol. Ther..

[73] Zhang E.J., Knox M., Veness M.J., Abdul-Razak M., Wong E., Hwang E.J., Carlino M., Sundaresan P. (2025). Outcomes with radiation therapy as primary treatment for unresectable cutaneous head and neck squamous cell carcinoma. Clin. Oncol. (R. Coll. Radiol.).

[74] Granger E.E., Kim E.Y., Karn E.E., Groover M.K., Silk A.W., Margalit D.N., Tishler R.B., Schoenfeld J.D., Ruiz E.S. (2024). Definitive radiation therapy for inoperable stage III/IV cutaneous squamous cell carcinoma: A single-institution retrospective cohort study. J. Am. Acad. Dermatol..

[75] Spelman L., Christie D., Kaminski A., Baker C., Supranowicz M., Sinclair R. (2022). Radiotherapy, utilizing volumetric modulated arc therapy, for extensive skin field cancerization: A retrospective case series assessing efficacy, safety, and cosmetic outcomes at 12 months after treatment. Case Rep. Dermatol..

[76] Park S.H., Ko H., Choi J. (2024). Effect of jaw tracking during volumetric modulated arc therapy for facial non-melanoma skin cancer. Vivo.

[77] Robinson A., Tallhamer M., Orman A. (2025). Optimizing breast cancer radiation therapy with volumetric modulated arc therapy and skin flash: A case study using deep inspiration breath hold and Cherenkov imaging. Adv. Radiat. Oncol..

[78] Zhang Y., Fu W., Brandner E., Percinsky S., Moran M., Huq M.S. (2024). Minimizing normal tissue low dose bath for left breast volumetric modulated arc therapy (VMAT) using jaw offset. J. Appl. Clin. Med. Phys..

[79] Teoh M., Clark C.H., Wood K., Whitaker S., Nisbet A. (2011). Volumetric modulated arc therapy: A review of current literature and clinical use in practice. Br. J. Radiol..

[80] Arunkumar T., Supe S.S., Ravikumar M., Sathiyan S., Ganesh M. (2010). Electron beam characteristics at extended source-to-surface distances for irregular cut-outs. J. Med. Phys..

[81] Reisner K., Haase W. (1990). Electron beam therapy of primary tumors of the skin. Radiol. Med..

[82] Mott J.H.L., West N.S. (2021). Essentials of depth dose calculations for clinical oncologists. Clin. Oncol. (R. Coll. Radiol.).

[83] Valve A., Koskenmies S., Tenhunen M., Nurmi H., Hernberg M., Salminen S., Anttonen A. (2023). Early clinical experience with a degraded 4 MeV electron beam in radiotherapy of superficial basal cell carcinoma. Phys. Imaging Radiat. Oncol..

[84] Rustgi S.N., Working K.R. (1992). Dosimetry of small field electron beams. Med. Dosim..

[85] Renard S., Parent L., de Marzi L., Tsoutsou P., Kirova Y. (2024). Electron radiation therapy: Back to the future?. Cancer Radiother..

[86] Zablow A.I., Eanelli T.R., Sanfilippo L.J. (1992). Electron beam therapy for skin cancer of the head and neck. Head Neck.

[87] Smits K., Quint K.D., Vermeer M.H., Daniëls L.A., Willemze R., Jansen P.M., Jansen W.P.A., Neelis K.J. (2022). Total skin electron beam therapy for cutaneous T-cell lymphomas in the Netherlands: A retrospective analysis of treatment outcomes and selection for high or low dose schedule. Clin. Transl. Radiat. Oncol..

[88] Esmati E., Abyaneh R., Jaberi R., Naderinasab S., Gholami S., Payandeh M., Salarvand F., Khalilian A., Seiri M., Lashkari M. (2024). Surface mold brachytherapy for head and neck non-melanoma skin cancer—Local control rates and survival: A retrospective analysis. J. Contemp. Brachyther..

[89] Fionda B., Placidi E., Rosa E., Lancellotta V., Stimato G., De Angeli M., Ciardo F.G., Cornacchione P., Siebert F.A., Tagliaferri L. (2023). Multilayer intensity modulated contact interventional radiotherapy (brachytherapy): Stretching the therapeutic window in skin cancer. J. Contemp. Brachyther..

[90] Benkhaled S., Van Gestel D., Cauduro C.G.S., Palumbo S., del Marmol V., Desmet A. (2022). The state of the art of radiotherapy for non-melanoma skin cancer: A review of the literature. Front. Med..

[91] Shah C., Ouhib Z., Kamrava M., Koyfman S.A., Campbell S.R., Bhatnagar A., Canavan J., Husain Z., Barker C.A., Cohen G.N. (2020). The American Brachytherapy Society consensus statement for skin brachytherapy. Brachytherapy.

[92] Tormo A., Celada F., Rodriguez S., Botella R., Ballesta A., Kasper M., Ouhib Z., Santos M., Perez-Calatayud J. (2014). Non-melanoma skin cancer treated with HDR Valencia applicator: Clinical outcomes. J. Contemp. Brachyther..

[93] Köhler-Brock A., Prager W., Pohlmann S., Kunze S. (1999). The indications for and results of HDR afterloading therapy in diseases of the skin and mucosa with standardized surface applicators (the Leipzig applicator). Strahlenther. Onkol..

[94] Delishaj D., Laliscia C., Manfredi B., Ursino S., Pasqualetti F., Lombardo E., Perrone F., Morganti R., Paiar F., Fabrini M.G. (2015). Non-melanoma skin cancer treated with high-dose-rate brachytherapy and Valencia applicator in elderly patients: A retrospective case series. J. Contemp. Brachyther..

[95] Zaorsky N.G., Lee C.T., Zhang E., Galloway T.J. (2018). Skin cancer brachytherapy vs external beam radiation therapy (SCRiBE) meta-analysis. Radiother. Oncol..

[96] Brovchuk S., Park S.-J., Shepil Z., Romanenko S., Vaskevych O. (2022). High-dose-rate skin brachytherapy with interstitial, surface, or a combination of interstitial and surface mold technique. J. Contemp. Brachyther..

[97] Ciurlia E., Santo B., Barba M.C., Cavalera E., De Franco P., De Matteis S., Di Paola G., Leone A., Papaleo A., Rubini D. (2025). Non-melanoma skin cancer treated with hypofractionated 192–Ir contact brachytherapy: A single institution series. Front. Oncol..

[98] Monge-Cadet J., Vairel B., Morisseau M., Moyal E., Ducassou A., Chira C., Pagès C., Sibaud V., Brun T., Modesto A. (2024). High-dose-rate brachytherapy for treatment of facial skin cancers: Local control, toxicity, and quality of life in 67 patients. Cancers.

[99] Oliveira C.N., Viveiros C., Travancinha C., Mota A., Pino I., Fonseca J., Madaleno T., Labareda M., Santos F., Esteves S. (2024). Treatment of basal cell carcinoma of the lower eyelid with high-dose-rate brachytherapy. Cureus.

[100] Bilski M., Cisek P., Baranowska I., Kordzińska-Cisek I., Komaniecka N., Hymos A., Grywalska E., Niedźwiedzka-Rystwej P. (2022). Brachytherapy in the treatment of non-melanoma skin peri-auricular cancers—A retrospective analysis of a single institution experience. Cancers.

[101] Renard S., Salleron J., Py J.F., Cuenin M., Buchheit I., Marchesi V., Huger S., Meknaci E., Peiffert D. (2021). High-dose-rate brachytherapy for facial skin cancer: Outcome and toxicity assessment for 71 cases. Brachytherapy.

[102] Virbel G., Ka K., Escande A., Mortier L., Barthoulot M., Liem X., Mirabel X., Lartigau E.F., Cordoba A. (2025). HDR and PDR brachytherapy for facial nonmelanoma skin cancer: Outcome and toxicity assessment for 155 patients. Brachytherapy.

[103] Bhatnagar A., Loper A. (2010). The initial experience of electronic brachytherapy for the treatment of non-melanoma skin cancer. Radiat. Oncol..

[104] Elekta AB–Nucletron Esteya^®^ Electronic Brachytherapy System. Stockholm, Sweden. http://www.esteya.com.

[105] Elekta Xoft Xoft^®^ Axxent^®^ Electronic Brachytherapy System. Nashua, NH, USA. https://www.elekta.com/products/brachytherapy/xoft/.

[106] Carl Zeiss Meditec AG INTRABEAM^®^ Electronic Brachytherapy System. Jena, Germany. https://www.zeiss.com/meditec/en/products/intrabeam-700.html.

[107] Eaton D.J. (2015). Electronic brachytherapy—Current status and future directions. Br. J. Radiol..

[108] Bhatnagar A. (2013). Nonmelanoma skin cancer treated with electronic brachytherapy: Results at 1 year. Brachytherapy.

[109] Garcia-Martinez T., Chan J.P., Perez-Calatayud J., Ballester F. (2014). Dosimetric characteristics of a new unit for electronic skin brachytherapy. J. Contemp. Brachyther..

[110] Cheng J., Henry G.V., Lyden M.R., Shrager D.I., Swann M.H., Stubbs J.B., Willard R.J., Lee E.K. (2024). The Elekta Esteya^®^ electronic brachytherapy system in non-melanoma skin cancers: A post-market observational study. J. Contemp. Brachyther..

[111] Doggett S.W., Willoughby M., Miller K.A., Mafong E. (2023). Long-term clinical outcomes of non-melanoma skin cancer patients treated with electronic brachytherapy. J. Contemp. Brachyther..

[112] Goyal U., Cheung M.K., Suszko J., Laughlin B., Kim Y., Askam J., Arif-Tiwari H., Slane B., Gordon J., Stea B. (2021). Electronic brachytherapy for treatment of non-melanoma skin cancers: Clinical results and toxicities. J. Contemp. Brachyther..

[113] Ballester-Sánchez R., Pons-Llanas O., Candela-Juan C., Celada-Alvarez F.J., de Unamuno-Bustos B., Llavador-Ros M., Ballesta-Cuñat A., Barker C.A., Tormo-Micó A., Botella-Estrada R. (2015). Efficacy and safety of electronic brachytherapy for superficial and nodular basal cell carcinoma. J. Contemp. Brachyther..

[114] Paravati A.J., Hawkins P.G., Martin A.N., Mansy G., Rahn D.A., Advani S.J., Hoisak J., Dragojevic I., Martin P.J., Miller C.J. (2015). Clinical and cosmetic outcomes in patients treated with high-dose-rate electronic brachytherapy for nonmelanoma skin cancer. Pract. Radiat. Oncol..

[115] Liu H., Chang J.Y. (2011). Proton therapy in clinical practice. Chin. J. Cancer.

[116] Lane S.A., Slater J.M., Yang G.Y. (2023). Image-guided proton therapy: A comprehensive review. Cancers.

[117] Bryant C.M., Dagan R., Holtzman A.L., Fernandes R., Bunnell A., Mendenhall W.M. (2021). Passively scattered proton therapy for nonmelanoma skin cancer with clinical perineural invasion. Int. J. Part. Ther..

[118] Yacoub I., Rayn K., Choi J.I., Bakst R., Chhabra A., Qian J.Y., Johnstone P., Simone C.B. (2024). The role of radiation, immunotherapy, and chemotherapy in the management of locally advanced or metastatic cutaneous malignancies. Cancers.

[119] Maillie L., Lazarev S., Simone C.B., Sisk M. (2021). Geospatial disparities in access to proton therapy in the continental United States. Cancer Investig..

[120] Patel P.V., Pixley J.N., Dibble H.S., Feldman S.R. (2023). Recommendations for cost-conscious treatment of basal cell carcinoma. Dermatol. Ther..

[121] International Atomic Energy Agency (2015). Implementation of High Dose Rate Brachytherapy in Limited Resource Settings.

[122] Kharrati K. (2024). Global Electronic Brachytherapy Market Size Likely to Reach at a CAGR of 9.48% by 2033.

[123] Particle Therapy Co-Operative Group Particle Therapy Facilities in Clinical Operation. Particle Therapy Co-Operative Group. https://www.ptcog.site/index.php/facilities-in-operation-public.

[124] Helgadottir H., Jönsson G., Wickström S., Nilsson J., Björkström K., Berglund A., Carneiro A., Ny L., Olofsson Bagge R. (2025). SWE-NEO: Swedish NeoAdjuvant Trial Comparing anti-PD-1 Monotherapy to Combined anti-CTLA-4/anti-PD-1 Blockade in Resectable Stage III Melanoma. Eur. J. Cancer Skin Cancer.

[125] Zandberg D.P., Allred J.B., Rosenberg A.J., Kaczmar J.M., Swiecicki P., Julian R.A., Poklepovic A.S., Schwartz G.K. (2025). Phase II (Alliance A091802) randomized trial of avelumab plus cetuximab versus avelumab alone in advanced cutaneous squamous cell carcinoma. J. Clin. Oncol..

[126] Nardone V., Napolitano S., Gagliardi F., Esposito A., Caraglia F., Briatico G., Scharf C., Ronchi A., D’Onofrio I., D’Ippolito E. (2024). Previous radiotherapy increases the efficacy of cemiplimab in the treatment of locally advanced and metastatic cutaneous squamous cell carcinoma: A retrospective analysis. J. Am. Acad. Dermatol..

[127] OncoBeta GmbH Rhenium-SCT^®^ Skin Cancer Therapy. Freiburg, Germany. https://www.oncobeta.com/our-products/rhenium-sct.

[128] Fukuda H. (2021). Boron neutron capture therapy (BNCT) for cutaneous malignant melanoma using ^10^B-p-boronophenylalanine (BPA) with special reference to the radiobiological basis and clinical results. Cells.

[129] Kashihara T., Nakamura S., Yamazaki N., Takahashi A., Namikawa K., Ogata D., Nakano E., Okuma K., Kaneda T., Mori T. (2025). Boron neutron capture therapy for cutaneous angiosarcoma and malignant melanoma: First in-human phase I clinical trial. Radiother. Oncol..

[130] Dziura D., Tabbassum S., MacNeil A., Maharaj D.D., Laxdal R., Kester O., Pan M., Kumada H., Marquardt D. (2023). Boron neutron capture therapy in the new age of accelerator-based neutron production and preliminary progress in Canada. Can. J. Phys..

[131] Durante M., Paganetti H. (2016). Nuclear physics in particle therapy: A review. Rep. Prog. Phys..

[132] Tsujii H., Mizoe J.E., Kamada T., Baba M., Kato S., Kato H., Tsuji H., Yamada S., Yasuda S., Ohno T. (2004). Overview of clinical experiences on carbon ion radiotherapy at NIRS. Radiother. Oncol..

[133] Blake P.R., Catterall M., Errington R.D. (1985). Treatment of malignant melanoma by fast neutrons. Br. J. Surg..

[134] Kawamura M., Kamomae T., Yanagawa M., Kamagata K., Fujita S., Ueda D., Matsui Y., Fushimi Y., Fujioka T., Nozaki T. (2024). Revolutionizing radiation therapy: The role of AI in clinical practice. J. Radiat. Res..

[135] Brancaccio G., Balato A., Malvehy J., Puig S., Argenziano G., Kittler H. (2023). Artificial intelligence in skin cancer diagnosis: A reality check. J. Investig. Dermatol..

[136] Venkatesh K.P., Kadakia K.T., Gilbert S. (2024). Learnings from the first AI-enabled skin cancer device for primary care authorized by FDA. NPJ Digit. Med..

[137] University Hospitals of Liverpool NHS Foundation Trust (2024). Merseyside Hospital Pioneers the Use of AI Technology for Early Detection of Skin Cancer.

[138] Horvat N., Papanikolaou N., Koh D.M. (2024). Radiomics beyond the hype: A critical evaluation toward oncologic clinical use. Radiol. Artif. Intell..

